# Lysosomal-Cleavable Peptide Linkers in Antibody–Drug Conjugates

**DOI:** 10.3390/biomedicines11113080

**Published:** 2023-11-16

**Authors:** Seetharamsing Balamkundu, Chuan-Fa Liu

**Affiliations:** School of Biological Sciences, Nanyang Technological University, 60 Nanyang Drive, Singapore 637551, Singapore; seetharamsing.b@ntu.edu.sg

**Keywords:** antibody–drug conjugate, lysosome, cathepsin, legumain, self-immolation, payload release, peptidomimetic, linkers

## Abstract

Antibody–drug Conjugates (ADCs) are a powerful therapeutic modality for cancer treatment. ADCs are multi-functional biologics in which a disease-targeting antibody is conjugated to an effector payload molecule via a linker. The success of currently used ADCs has been largely attributed to the development of linker systems, which allow for the targeted release of cytocidal payload drugs inside cancer cells. Many lysosomal proteases are over expressed in human cancers. They can effectively cleave a variety of peptide sequences, which can be exploited for the design of ADC linker systems. As a well-established linker, valine-citrulline-*p*-aminobenzyl carbamate (ValCitPABC) is used in many ADCs that are already approved or under preclinical and clinical development. Although ValCitPABC and related linkers are readily cleaved by cathepsins in the lysosome while remaining reasonably stable in human plasma, many studies have shown that they are susceptible to carboxylesterase 1C (Ces1C) in mouse and rat plasma, which hinders the preclinical evaluation of ADCs. Furthermore, neutropenia and thrombocytopenia, two of the most commonly observed dose-limiting adverse effects of ADCs, are believed to result from the premature hydrolysis of ValCitPABC by human neutrophil elastase. In addition to ValCitPABC, the GGFG tetrapeptidyl-aminomethoxy linker is also cathepsin-cleavable and is used in the highly successful ADC drug, DS8201a. In addition to cathepsin-cleavable linkers, there is also growing interest in legumain-sensitive linkers for ADC development. Increasing plasma stability while maintaining lysosomal cleavability of ADC linkers is an objective of intensive current research. This review reports recent advances in the design and structure–activity relationship studies of various peptide/peptidomimetic linkers in this field.

## 1. Introduction

**Figure 1:**
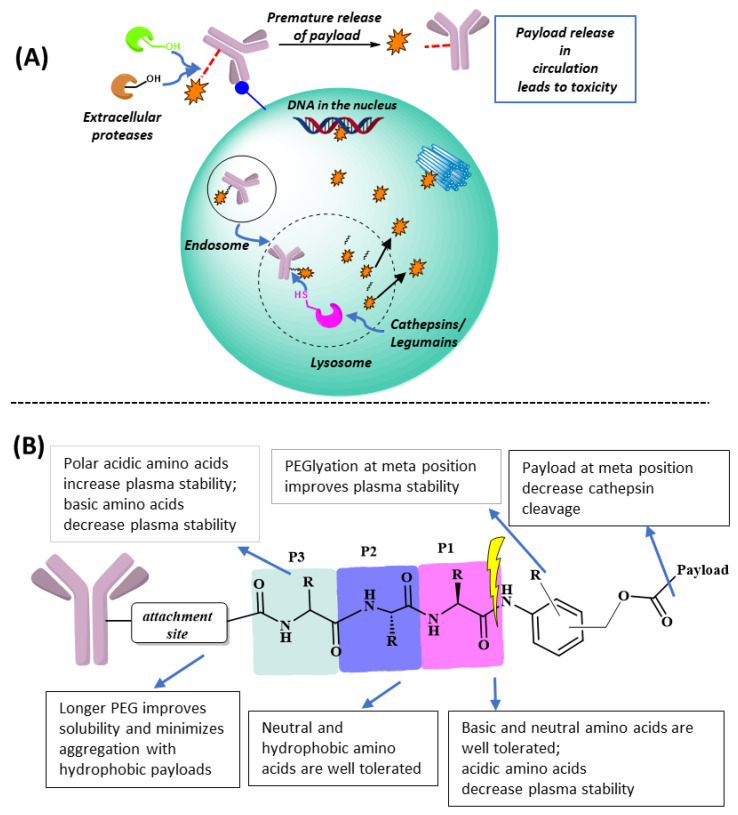
(**A**) Fate of an ADC before and after internalization. Premature cleavage of the linker in extracellular matrix is often associated with off-target toxicity. Antigen-mediated endocytosis delivers ADC in the endosomal–lysosomal system and lysosomal linker cleavage releases the drug, which acts to exert its cytotoxicity. (**B**) Effect of amino acid composition in the linker peptide and substitution on the benzene ring of PABC on linker stability in mouse plasma.

Clinical approvals of Antibody–Drug Conjugates (ADCs) have seen a big leap in the past several years. In fact, 12 out of 15 ADCs on the market were approved from 2017 to 2022 [1,2]. More than 100 new ADCs are currently at various stages of clinical development, which reflects the huge potential of this class of medicines in cancer treatment. The mechanism of action of ADCs involves antibody binding to a specific antigen on cancerous cells and subsequent internalization via receptor-mediated endocytosis. Once inside the cancer cell, degradation of the antibody and/or cleavage of the linker in the endosomal–lysosomal compartments would release the drug payload, which then exerts its cytocidal effects in the cytoplasm or nucleus. Researchers have taken advantage of two important endosomal–lysosomal features in designing linker systems for drug release: (i) the acidic environment inside lysosomes for the design of acid-labile linkers, and (ii) the over-expression of specific lysosomal proteases for the design of protease-cleavable linkers (Figure 1A) [3,4,5,6,7,8,9]. Eight out of the fifteen approved ADCs have protease-recognizable peptide sequences in the linkers (Table 1), which are further attached to a self-immolated moiety. Upon cleavage of the peptide sequence by the protease, the self-immolated moiety readily undergoes elimination to release the free drug, which then defuses out of the lysosomal compartment. Linker–payload optimization is one of the most critical tasks in ADC development. The lysosomal protease-cleavable Valine-Citrulline-PABC (ValCitPABC) linker system is used in many of the approved ADCs [10,11], which is readily cleaved at the amide bond linking Cit and PABC, leading to self-immolate payload release. Initially, it was thought that only cathepsin B was responsible for the cleavage of ValCitPABC; however, later gene knockout studies have shown that other cathepsins, like cathepsin S, cathepsin L, and cathepsin F, are also involved in the cleavage mechanism [12]. It has also been revealed that a variety of dipeptide sequences can act as substrates for these lysosomal enzymes. After the initial development of the ValCitPABC linker, researchers have tested a plethora of peptide/peptidomimetic sequences to further improve the linker system [2,3,4,5,7,13] for faster release of payloads and to discover more enzyme-specific peptide sequences, which led to the identification of new cathepsin-sensitive dipeptide sequences such as ValAla, AlaAla, and cBuCit (cBu: cyclobutane-1,1-dicarboxamide). 

The ValCitPABC linker system has proven to have good stability in human serum, which is the utmost criterion for ADCs to sustain enzymatic degradation in systemic circulation. However, many studies have shown that ValCitPABC is susceptible in mouse plasma and that the degradation is caused by a protease called Carboxylesterase C1 (Ces1C) [14]. This instability in mouse plasma hampers the preclinical evaluation of the ADCs. Therefore, the preclinical studies have to be conducted in transgenic mice with knock-out Ces1C. Target-independent uptake toxicity [15], dose-limiting neutropenia, and thrombocytopenia [3,4,5,6,8,16] are major causes of concern in ADC development. It has been shown that ADC treatment often leads to neutropenia in cancer patients [17], especially when employing ValCitPABC-MMAE. Zhao et al. [17] employed purified neutrophil elastase, a serine protease, to evaluate the in vitro stability of ADCs with a cleavable valine-citrulline linker (vc-MMAE), or a non-cleavable maleimidocaproyl linker (mc-MMAF), and showed that the ValCit linker was readily cleaved by elastase to release free MMAE, whereas the MC linker was not. 

A number of recent review articles have covered ADC linkers designed on cleavage mechanisms by lysosomal enzymes like cathepsins, phosphatases, pyrophosphatases, sulfatases, β-galactosidase, and β-glucuronidase, as well as via the reduction of disulfide linkages by glutathione [18,19,20,21,22]. In this mini review, we attempt to capture the most recent developments in the design of peptide linkers with improved selectivity towards specific cleavage enzymes in lysosomes and increased stability in plasma. 

## 2. Adding a P3 Polar Acidic Residue to the ValCitPABC Linker Increases Plasma Stability

The ValCitPABC linker is designed to be cleaved by cathepsin B at the amide bond between the P1 residue citrulline and P1′ PABC (Figure 1B) [10]. Cathepsin B, a cysteine protease primarily found in lysosomes, is first synthesized as a pre-proenzyme in the endoplasmic reticulum. Upon removal of the N-terminal signal peptide, the proenzyme is delivered to the Golgi apparatus, where it is modified via glycosylation. The glycosylated proenzyme then translocates from the trans-Golgi network to endo/lysosome, where auto-proteolytic processing converts it into the mature form composed of two disulfide-linked polypeptide chains [23]. Like other lysosomal cathepsins, cathepsin B is often upregulated in cancer cells [24,25], which makes the ValCitPABC linker particularly attractive for ADC payload release. However, it has been shown that ValCitPABC can be cleaved by Ces1C in mouse plasma, as verified with the purified Ces1C enzyme and the Ces1C knockout mouse as the positive and negative controls, respectively [14]. In the same study, the ValCit linker was found to be stable when co-incubated with Ces1C inhibitors. After clearly establishing the Ces1C-mediated cleavage mechanism, the authors synthesized several novel linker–payload molecules with a substitution at the P3 position and tested their stability (Figure 1B). Interestingly, they observed increased mouse plasma stability when certain hydrophilic groups were introduced at the P3 position; in particular, a 2-hydroxy acetamide group greatly increased the plasma stability. Inspired by these results, Anami et al. introduced hydrophilic amino acids at the P3 position [26] and found that, while SerValCit showed little improvement in stability, linkers with an acidic amino acid at the P3 position, GluValCit and AspValCit, showed excellent stability in mouse plasma. In contrast, having a basic amino acid Lys at P3 made the LysValCit linker more labile than the parent ValCit linker. This indicates that an acidic amino acid at the P3 position effectively blocks the access by Ces1C, whereas a basic amino acid enhances the interaction between Ces1C and the linker. Importantly, all the linker–payload designs discussed above were very stable in human serum and they remained susceptible to the lysosomal enzyme cathepsin B, which is crucial for drug release inside the cancer cell, suggesting that lysosomal cleavage enzymes do not have stringent sequence specificity. It is hypothesized that the human homolog of the Ces1C enzyme in human liver has the active-site serine residue deep inside the substrate binding cleft [14], whereas the active site of human cathepsin B is shallow; thus, substituents at the P3 site are well tolerable. 

## 3. Polar Basic Residue Substitution at the P1 Position of the ValCit-PABC Linker Improves Lysosomal Cleavage Activity

Since its initial discovery, the ValCit-PABC linker system has been used in many ADC constructs with a variety of antibodies and payloads. Researchers have designed a plethora of P2-P1 dipeptides via the substitution of P2-Val and P1-Cit, aiming to increase the linker’s lysosomal cleavability and improve its stability in mouse plasma. Poudel et al. synthesized various linkers with uncialamycin as the payload [27]. When the polar citrulline was replaced with alanine, the stability in plasma was further decreased, although payload release by cathepsin B was not affected. When the polar negatively charged aspartic acid was introduced at the P1 position, there was no significant change in mouse plasma stability; however, it showed a decreased release of the payload by cathepsin B. On the contrary, another study on the effect of the P1 amino acid on cathepsin-mediated cleavage showed that an arginine residue at the P1 position improved the cleavage by nine fold [28]. These results suggest that a polar or basic P1 amino acid is preferable for efficient payload release, whereas an acidic residue decreases the cleavage efficiency. 

## 4. Peptidomimetic Substitution on the Cathepsin-Specific Dipeptide-PABC Linker

**Figure 2:**
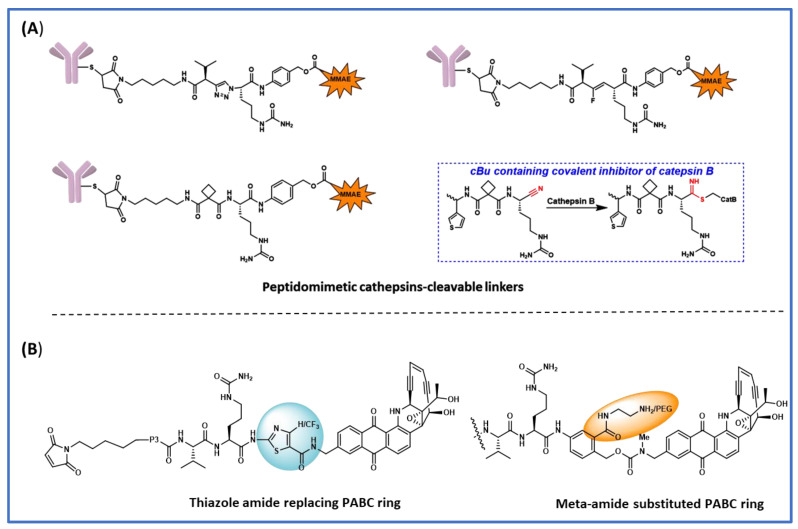
Various Linker payload designs for faster lysosomal cleavage and improved plasma stability. (**A**) Peptidomimetic linkers specifically cleaved by cathepsin B; (**B**) modifications to central PABC ring.

**Figure 3:**
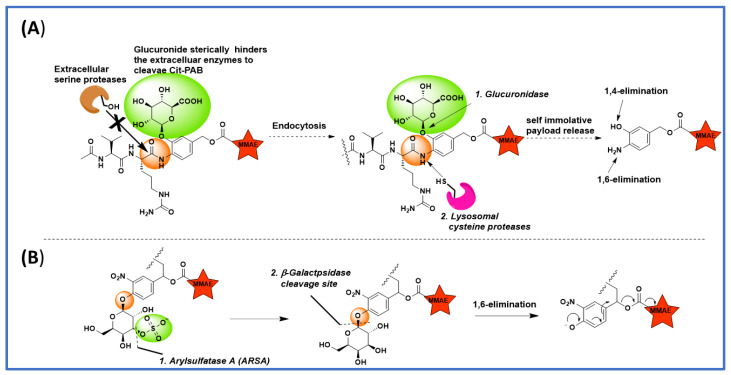
Various Linker payload designs for faster lysosomal cleavage and improved plasma stability. (**A**) Tandem cleavable linkers’ glucuronide group masks the linker system to maintain stability in extracellular environment; (**B**) Aryalsulfatase A (ARSA) and β-galactosidase dual cleavable 3-*O*-sulfo-β-galactose linker.

In a structure-based study of novel peptidomimetic substitutions at the P2 position of the ValCit-PABC linker, Wei et al. [28] took the key assumption that reducing the number of hydrolyzable peptide bonds would yield cathepsin B-specific linkers with improved extracellular stability [28]. In this study, they used simplified constructs where linkers were attached to norfloxacin via PABC and the N-terminal was protected as benzyl carbamate. Cleavage efficiency was evaluated using the Michaelis–Menten steady state *V*_max_ and the *K*_m_ data by keeping the cathepsin B concentration constant. When they replaced the P2-P1 peptide bond with fluoroolefin and triazole, the cleavage activity by cathespsin B was greatly reduced, which suggests hydrogen bond interactions are crucial for enzyme binding. Based on computational shape similarity search, they identified cyclobutane-1,1-dicarboxamide as a suitable P2 residue. This novel structural unit is able to provide three hydrogen bonding interactions and the cyclobutyl group has an optimum size to fit in the S2 binding pocket. They synthesized various linkers with varying sizes of cycloalkyl rings and side chains at the P1 site; however, none of these showed increased cleavage activity compared to the ValCit counterpart. To address the discrepancies between the computational model and the observed cleavage results, they obtained a crystal structure of human cathepsin B complexed with thiophen-cBuCit-CN (Figure 2A), where the electrophilic CN group was introduced to capture the Cys thiol group at the active site. Indeed, the crystal structure showed expected hydrogen bonding interactions and the key thioimidate bond formed between the cysteine and C≡N and the cyclobutyl group perfectly sitting in the S2 binding pocket. Encouraged from the crystal structure results, they went on to synthesize the ADCs, anti-Her2-mc-cBuCitPABC-MMAE, and anti-Her2-mc-ValCitPABC-MMAE (Figure 3B), using an engineered cysteine strategy with DAR between 1.8 and 2.0. When these ADCs were evaluated against Her-2-expressing SKBR-3 cells, both ADCs showed almost similar antigen dependent anti-cancer activity and were equally stable in the in vivo mouse models. When incubating the ValCit and cBuCit ADCs with inhibitors of cathepsin B, they found that cBuCit cleavage activity was reduced by 90% vs. 50% for ValCit, demonstrating the high specificity of the cBuCit linker towards cathepsin B. 

## 5. The Effect of Substitution on the PABC Benzene Ring 

Directly attaching Val-Cit to the payload leads to a less efficient payload release due to the steric factors of the payload, which inhibits cathepsin binding to the ValCit dipeptide. This problem is partially solved when a spacer is added between the payload and ValCit. PABC (*para-aminobenzyl carbamate*) not only improves cathepsin binding, but it also undergoes self-immolative 1,6-elimination to release the payload in the unmodified form. Currently, PABC is used with a variety of payloads and peptide linker systems. To improve the stability of the ValCit-PABC linker system toward Ces1C in mouse plasma, Podule et al. synthesized several uncialamycin-linker conjugates [27,29] with substitutions on or the replacement of PABC, including replacement by heterocycles like thiazole. These conjugates were prepared from the reaction of MC-peptide-PABC-uncialamycin linkers and N-acetyl cysteine. They determined the percentage of the drug released after 24 h incubation with cathepsin B, human serum, and mouse serum. Introducing a thiazole amide group in place of PABC decreased the cleavage in mouse serum; however, cleavage in human serum was also observed. When an electron-withdrawing CF_3_ group was introduced on the thiazole ring, the cleavage in mouse serum was further decreased, and so was the cleavage in human serum. Combining the CF_3_-substituted thiazole with an aspartic acid at the P3 position generated a linker with only 6% and 4% cleavage in mouse and human serum after 24 h incubation. It is noteworthy that all of these PABC linker analogues were cleaved 100% by cathepsin B, which indicates that the modifications are well tolerated by cathepsin B. Next, they tried to see the impact of substitutions on the PABC benzene ring. When ValCit was attached via ortho allylation to benzyl alcohol, another possible position for self-immolation, cathepsin cleavage activity was completely lost due to the high steric hindrance of the ortho substitution. Furthermore, the linker did not survive in mouse serum. Encouraging results were obtained when an *N*-methyl carboxyamide group was introduced at the meta position. The resultant MA-PABC was only 3% cleaved in mouse serum after 24 h incubation and its cathepsin-mediated release was not affected. When a glutamic acid was added at the P3 position to the same MA-PABC, the mouse serum cleavage was further reduced to 7% over 24 h. Adding a glutamic acid not only improves the stability towards Ces1C but also the solubility, which is especially important when hydrophobic payloads are used. The authors went further to increase the hydrophilicity of linkers by replacing the methyl group with 2-aminoethyl and its amino-PEGylated form (Figure 2B). These new modifications provided the linkers with excellent mouse and human serum stability without compromising the cathepsin B-mediated cleavage. The ADCs generated from the above PEG-linker–payload using bacterial transglutaminase chemistry showed antigen-dependent activity without problems of linker hydrolysis in mouse serum. The above study clearly demonstrates that combining various attributes from structure–activity relationship studies can yield ideal linker–payload systems with high stability in plasma while maintaining cathepsin susceptibility. 

## 6. Tandem Cleavable Linkers

A common drawback of ValCitPABC-linked ADCs is myelosuppression, which is most likely caused by premature linker cleavage and payload release. In an effort to improve the in vivo stability of ValCitPABC-MMAE-conjugated ADCs, Chuprakov et al. developed an interesting tandem cleavable linker [30]. They incorporated a β-glucuronide moiety onto PABC, which would act as a steric blocker to protect the ValCitPABC linker from serine proteases in circulation (Figure 3A). After internalization, the β-glucuronidase-mediated cleavage of β-glucuronide would first unmask ValCitPABC, which would then be readily cleaved by cathepsins to release MMAE inside the cancer cells. An anti-CD79b antibody was used for conjugation with the tandem linker-MMAE payload using Hydrazino-iso-Pictet-Spengler (HIPS) chemistry between an aldehyde and a hydrazino-indole [31] to afford an ADC with DAR1.7 [32]. An ADC with an average DAR of 3.2 was also prepared using conventional MC-ValCitPABC-MMAE via cysteine conjugation for direct comparison. They synthesized several ADCs with the β-glucuronide moiety incorporated at the P1′ PABC position (Figure 3A). All the ADCs were equally potent when tested in vitro against B-cell non-Hodgkin lymphoma-derived CD79b+ JeKo-1 cells. As expected, the conventional ValCitPABC-MMAE ADC lost 20% of the payload when incubated in rat plasma at 37 °C for one week, whereas no payload loss was observed in ADCs with the tandem cleavable linker. When tested in mice carrying Granta 519 xenografts, an ADC prepared from cysteine-conjugated MC-ValCit-PABC-MMAE with a β-glucuronide moiety on P1′ PABC showed almost equal potency in reducing the tumor volume. Interesting results were observed from in vivo tolerability and toxicokinetic studies in which the ADCs were dosed to Sprague−Dawley rats. Clinical chemistry and hematology were assessed on day 5 and day 7. Key indicators of myelosuppression, namely the levels of monocytes, neutrophils, and eosinophils, were studied. Rats treated with the conventional mono-cleavage-linker ADCs had marked reductions in the levels of circulating monocytes, neutrophils, and eosinophils, whereas rats treated with tandem-cleavage-linker ADCs did not show any evidence of myelosuppression. In fact, the levels of circulating monocytes, neutrophils, and eosinophils of the latter group were similar to those in vehicle control groups, indicating clearly that masking with the β-glucuronide moiety at P1′ greatly increased linker stability in circulation. To further confirm it, plasma samples were taken at various time points and analyzed with ELISA methods, of which rapid payload loss was found in the mono-cleavage ADCs as opposed to tandem-cleavage ADCs. The released payload would be the likely reason for the observed myelosuppression. Although the improved safety of the tandem cleavable linkers was demonstrated in studies with rodent models only, they are likely to have similar beneficial effects in humans because the steric hindrance around the Cit-PABC amide bond would also enhance the resistance toward any circulating human proteases. 

In addition to plasma instability, the dipeptide-based linkers are often hydrophobic in nature, which may cause ADC aggregation. To address these issues, Bargh et al. reported a dual enzyme-cleavable 3-*O*-sulfo-β-galactose linker [33] (Figure 3B), which can be cleaved in a cascade reaction via the consecutive actions of arylsulfatase A (ARSA) and β-galactsedase (β-gal). To validate this concept, they synthesized the 3-*O*-sulfo-β-galactose linker with 7-amino-4-methyl coumarin (AMC) as a model payload, incubated it with ARSA, β-gal, and a mixture of both ARSA and β-gal, and measured the fluorescence signal of the released AMC. As anticipated, incubation with either ARSA or β-gal alone did not produce any fluorescence signal, but incubation with both enzymes gave dramatic fluorescence, indicating that both enzymes were needed for the payload release. This ARSA/β-gal combination greatly improved the lysosome-selective cleavage. Further, the presence of an anionic sulfate group and a galactose moiety greatly improved the solubility of the linker in the aqueous medium. Having encouraging results in fluorescence studies, they synthesized 3-*O*-sulfo-β-galactose-MMAE linker payloads with bis-thiol reactive divinylprimidine (DVP), which was conjugated with reduced α-HER2-trastuzumab to afford ADCs with DAR4. The generated ADCs were tested against HER2-positive SKBR3 and HER2-negative MFC7 cells. Excellent potency (IC_50_ = 49 pM), which is comparable to Val-Ala-PABC-MMAE (IC_50_ = 41 pM), and selectivity were observed. The 3-*O*-sulfo-β-galactose linker system would be useful in generating ADCs, having highly hydrophobic payloads which are otherwise known to aggregate easily in an aqueous medium.

## 7. The GGFG Tetrapeptidyl-Aminomethoxy Linker 

**Figure 4:**
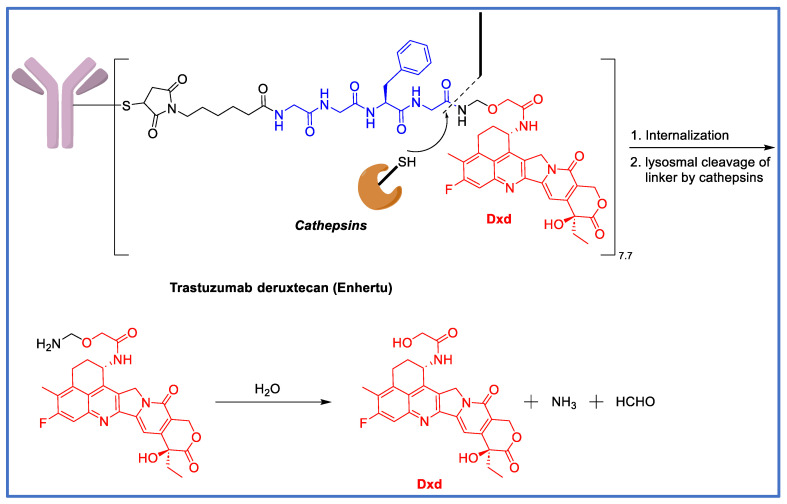
Structure and linker cleavage mechanism of trastuzumab deruxtecan (Enhertu).

In addition to the popular peptidyl-PABC system, another peptide-based linker, which contains an aminomethoxy group for self-immolative drug release, is used in the successful development of trastuzumab deruxtecan (Enhertu^®^) (Figure 4). In 1995, Nogusa et al. prepared the conjugates of doxorubicin by attaching it, via its amino group, to carboxymethylpullulan [34]. They demonstrated that free doxorubicin was effectively released by lysosomal enzymes when a tetrapeptide spacer, GGFG, was placed between the drug and the polysaccharide polymer, suggesting that the amide linkage between the C-terminal glycine in the spacer and the amino group of doxorubicin was enzymatically cleaved. When the peptide spacer was GFGG, doxorubicin could still be released, but to a much lesser degree than the GGFG spacer. The conjugate without a peptide spacer had no detectable drug release. They also showed that the anti-tumor activity of the conjugates paralleled the cleavability of the linker, with the GGFG conjugate being the most active and the no-spacer conjugate the least active [34]. In a study published in 2007 [35], Shiose et al. reported a macromolecular prodrug of DX8951 (exatecan) [36], in which the polymer carrier, carboxymethyldextran polyalcohol (CM-Dex-PA), was coupled to exatecan at its amino group via the GGFG tetrapeptide spacer. It was found that cathepsins B, H, and L from lysosomes were able to cleave the glycinyl–exatecan amide bond to release free exatecan. Building on this, Ogitani et al. developed a Her-2-targeting ADC, DS8201a (the code name of transtuzamab-deruxtecan), with DXd (an analog of exatecan, i.e., hydoxylacetyl-exatecan) as the payload [37]. DS8201a is generated using cysteine–maleimide chemistry with a theoretical DAR of 8. The hydroxyl group in DXd is utilized for antibody attachment via the maleimide-functionalized GGFG-aminomethoxy linker. After ADC internalization, cathepsins cleave the amide bond between the C-ter glycine of GGFG and the aminomethoxy group, triggering the decomposition of the latter to release free DXd (Figure 4). DS8201a showed good stability in mouse, rat, or human plasma with only a 1–2% drug release over 21 days [37]. In a subsequent study [38], Ogitani et al. found that DS8201a exhibited an excellent bystander effect because, with the free amine in exatecan capped by hydroxyacetyl, the DXd payload cannot be ionized via protonation, and thus, has high membrane permeability. DS8201a was also found to be a very effective ADC against Her-2-low-expressing cancers [39], which is mainly attributed to a high DAR of 8 and the by-stander effect of DXd by diffusing through the cell membrane and killing nearby cancer cells. 

## 8. Legumain-Cleavable Linkers

**Figure 5:**
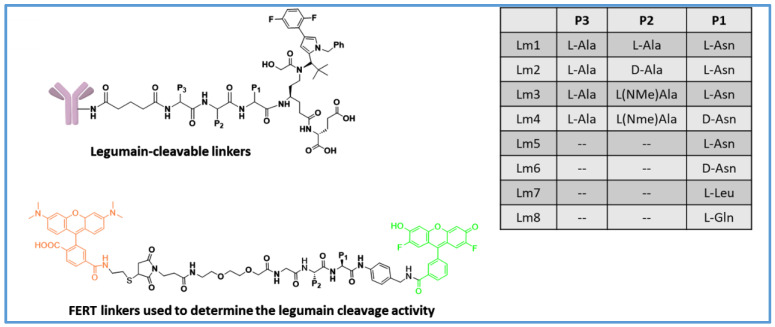
Asn-containing peptide linkers with improved selectivity towards legumain.

Legumain, an asparaginyl endopeptidase [40,41,42], is known to have high expression levels in cancer cells. Like cathepsins, it is also present in lysosomes. Legumain is first synthesized as a pro-enzyme and an acidic pH (pH 4–5) is needed for its activation. It has increased expression in solid tumors, where it plays a key role in tumor invasion and metastasis [43], which makes it an attractive alternative to cathepsins for the development of protease-cleavable linkers [44]. Bajjuri et al. found that the tripeptide-conjugated MMAE, AlaAlaAsn-PABC-MMAE, was a prodrug to MMAE, which is otherwise too toxic to use as a free drug [45]. It was shown that AlaAlaAsn-PABC-MMAE was effectively cleaved by legumain and was less toxic than free MMAE in mouse models. Cheng et al. used the cysteine conjugation approach to generate several ADCs with different cleavable linkers, with varying lengths of PEG as the spacer and microtubule inhibitors as the payloads. They showed that an ADC prepared using the polyethylene glycol-extended legumain-cleavable tripeptide linker payload system, PEG2-AlaAlaAsn-PABC-eribulin, showed similar cytotoxicity when compared to other ADCs prepared from other linkers [46]. However, they did not disclose the in vivo efficacy data in the mouse models. Lerchen et al. reported rather interesting results on legumain-cleavable linkers [47]. They prepared TWEAKR-targeting ADCs with a kinesin spindle protein inhibitor (KSPi) as the payload [48,49]. The KSPi has a primary amine group, which was directly attached to the peptide linker without a self-immolative system. The alpha carbon of the KSPi primary amine was functionalized with a propionyl-Glu moiety (Figure 5) to modulate the physiochemical properties of the active metabolite as the polar glutamate reduced the permeability, thus enabling longer retention inside the tumor cells. They synthesized constructs with varying linker sequences, such as L-Ala-L-Ala-L-Asn (Lm-1), L-Ala-D-Ala-L-Asn (Lm-2), L-Ala-L(*NMe*)Ala-L-Asn (Lm-3), L-Ala-L-(*NMe*)Ala-D-Asn (lm-4), L-Asn (Lm-5), D-Asn (Lm-6), L-Leu (Lm-7), and L-Gln (Lm-8) (Figure 5), and evaluated in vitro protease cleavage activity with legumain, cathepsin B, and neutrophil elastase. As anticipated, D-Asn-containing constructs and constructs with a single Asn showed very poor hydrolysis activity by legumain. All the constructs showed no cleavage with cathepsin B and neutrophil elastase. Surprisingly, when the corresponding ADCs were incubated with NCI-H292 cells, for ADCs with linkers Lm-2 and Lm-5, similar amounts of active metabolite was observed in the cell lysate, indicating the expression of high levels of legumain in tumor cells. In in vitro efficacy studies against the TWEAKR-expressing NCI-H292 lung cancer, BxPC3 pancreatic cancer, and LoVo colon cancer cell lines, the Lm-1, Lm-2, and Lm-3 ADCs showed excellent potency. Replacing the central L-Ala with D-Ala did not affect the activity; however, when the L-Asn was replaced with unnatural D-Asn, the activity was completely lost. The most intriguing results were that of the ADC with a single L-Asn in the linker, which retained the activity. Similar activity trends were observed with Her-2-targeting ADCs. Liver toxicity of the ADCs were evaluated by incubating in the lysosomal preparation of rat liver for 48 h using the classic ValCit-PABC ADC as a positive control, which showed an 85% cleavage; however, the new ADCs with legumain-cleavable linkers were found to be much more stable with little hydrolysis: 18% for Lm-1, 5% for Lm-3, and less than 1% for Lm-2 and Lm-5. Excellent in vivo anti-tumor activity was observed with these ADCs when treated with mice bearing NCI-H292 and Ku-19-19 tumors. 

In a high throughput enzyme agnostic screening, Miller et al. used a Förster resonance energy transfer (FRET) assay to study cleavage efficiency in lysosomal extracts and stability in human and mouse plasma of 75 peptide FERT pairs (Figure 5) [50]. They found that various Asn-containing dipeptide sequences were readily cleaved by legumain. In enzyme-based assays, peptide sequences, like AsnAsn, AsnAla, were selectively cleaved by legumain and very stable towards cathepsin B. The newly identified Asn peptide linkers were found to be very stable in mouse plasma and resistant to Ces1c-mediated cleavage. They synthesized anti-Her2 and anti-CD20 ADCs with the AsnAsn, GlnAsn, AlaAsn, ValCit dipeptide-PABC-MMAE linker–payload system, using cysteine–maleimide chemistry for conjugation (DAR~6). In vitro cytotoxicity of the ADCs was evaluated using Her-2-expressing SKBR3 cells and CD20-expressing Ramos cells. Compared with the cathepsin-cleavable ValCit ADCs, the legumain-cleavable ADCs showed similar activities; however, Asn-containing ADCs showed 2–3-fold higher off-target activity than the ValCit ADC. The reduced selectivity of legumain-cleavable ADCs is likely because that legumain is also secreted in the tumor microenvironment, and thus, might have caused extracellular linker cleavage. 

## 9. Site of Conjugation on ADC Stability and Activity

In addition to the linker, the conjugation site can also have a significant influence on the stability of an ADC during circulation, and hence, its overall pharmacological activity. The availability of many different site-specific conjugation methods [51] has made it possible to choose suitable sites on the antibody for payload attachment. The thiol–maleimide Michael addition chemistry is a commonly used method because of its robustness and chemo-specificity. The reaction leads to a succinimide thioether linkage formed with either native or engineered cysteine residues in the antibody. However, the maleimide exchange with thiols present in certain plasma components can result in deconjugation, which negatively affects ADC efficacy and safety. The succinimide ring can also undergo hydrolysis to produce a stable linkage which cannot further undergo retro-Michael elimination and is therefore beneficial for ADC activity. Shen et al. have shown that the different solvent accessibility and chemical environment of different conjugation sites can have different effects on the above-mentioned two reactions, which in turn affects the stability and efficacy of the corresponding ADCs [52]. It is interesting to note that conjugation site-dependent payload loss in plasma was observed not only with the ValCit-PABC linkers, but also with the non-cleavable linkers. The results from this study seem to show that the decrease in DAR was due to chemical deconjugation; however, one may not completely exclude the possibility that the enzymatic cleavage of the ValCit-PABC linker also played a role, though likely to a much lesser extent. Bioconjugation using microbial transglutaminase (mTG) has been used to assess how the stability of the antibody–drug linker is influenced by the site of the conjugation. In a study conducted by Dorywalska et al., it is shown that the conjugation site critically affects the enzymatic susceptibility of the ValCit-PABC linker to unidentified serine proteases in mouse plasma (likely Ces1C), of which there is a positive correlation between linker stability and ADC cytotoxic potency both in vitro and in vivo [53]. In another study [54], Kaempffe et al. generated three trastuzumab-derived ADCs using the Val-Cit-PABC linker via mTG conjugation at native Q295 and two engineered glutamine sites at the C-terminus of heavy or light chains. They found that, in huFcRn transgenic mice, the Q295- or light chain C-terminal-conjugated ADC displayed a superior PK property, whereas the heavy chain C-terminal-conjugated ADC showed faster clearance. Therefore, whilst linker design is an important consideration, a holistic approach is to also take into account, among other things, the site of the conjugation in ADC development. As seen from the studies discussed here, a carefully chosen conjugation site can provide a favorable local environment to protect the conjugate and the linker against chemical and/or enzymatic attacks during systemic circulation.

## 10. Conclusions and Outlook

The immense research effort put into ADC linker–payload design during the past decade has contributed to a sharp increase in regulatory approvals. However, ADC development still faces dose-limiting toxicity issues during clinical trials, which could be partly attributed to the systemic release of the payload and poor PK properties. The development of more stable linker systems in mouse and rat plasma would accelerate the preclinical evaluation of the ADC drug candidates. In particular, the identification of new peptide/peptidomimetic sequences will allow highly specific cleavage, which can greatly reduce premature cleavage of the linker at non-tumor sites, hence increasing the safety profiles of the ADCs. Obviously, the linker systems reviewed herein apply only to typical ADCs which rely on binding to an internalizing antigen and subsequent receptor-mediated endocytosis to release the payload via lysosomal degradation. Nevertheless, recent years have also seen growing interest in developing an atypical class of ADCs targeting poorly- or non-internalizing antigens on cancer cells or components of surrounding tumor stroma. For these ADCs, the linkers are cleaved extracellularly within the tumor microenvironment to release the payload, which can then diffuse into nearby cancer cells for selective killing. However, the design of such linker systems is beyond the scope of the current review. The readers are advised to refer to an excellent recent review article by Ashman et al. for a comprehensive account on this topic [55]. In addition to linker design, site-specific conjugation using engineered antibodies and enzyme systems now enables the development of homogeneous ADCs with superior PK properties. The use of a site-specific conjugation method also allows for payload attachment at sites that can enhance systemic stability of the prepared ADCs. The use of improved linker–payload designs and highly active payloads in combination with site-specific conjugation chemistry will lead to more successes in the ADC field in the future.

## Figures and Tables

**Table 1:** Clinically approved ADCs.

S. No.	ADC	Linker System	Cleavage Mechanism	Payload	Company
1	Gemtuzumab ozogamicin (Mylotarg)	4-(4-acetylphenoxy) butanoic acid	pH sensitive	Calicheamicin	Pfizer/Wyeth
2	Brentuximab vedotin (Adcetris)	mc-ValCitPABC	Lysosomal	MMAE	Seattle/Takeda
3	Trastuzumab emtansine (Kadcyla)	MCC	Non-cleavable	Maytansine DM1	Genentech Roche
4	Inotuzumab ozogamicin (Besponsa)	Hydrazone	pH sensitive	Calicheamicin	Pfizer/Wyeth
5	Polatuzumab vedotin (Polivy)	mc-ValCitPABC	Lysosomal degradation	MMAE	Genentech Roche
6	Enfortumab vedotin (Padcev)	mc-ValCitPABC	Lysosomal degradation	MMAE	Astellas/Seattle Genetics
7	Trastuzumab deruxtecan (Enhertu)	mc-GGFG-aminomethoxy	Lysosomal degradation	Deruxtecan, Dxd	Daiichi-Sankyo/AstraZeneca
8	Sacituzumab govitecan (Trodelvy)	mc-PEG-carbonate	pH	SN-98	Immunomedics
9	Belantamab mafodotin (Blenerp) *	mc-MMAF	Non-cleavable	MMAF	GSK
10	Loncastuximab tesirine (Zynlonta)	mc-ValCitPABC	Lysosomal degradation	SG3199, PDB dimer	ADC Therapeutics
11	Tisotumab vedotin (Tivdak)	mc-ValCitPABC	Lysosomal degradation	MMAE	Genmab and Seattle Genetics
12	Disitamab Vedotin (Aidixi)	mc-ValCitPABC	Lysosomal degradation	MMAE	RemeGen
13	Moxetumomab pasudotox (Lumoxiti)	mc-ValCitPABC	Lysosomal degradation	PE38	AstraZeneca
14	Cetuximab sarotalocan (Akalux)	NA	NA	IRDye700DX	Rakuten Medical
15	Mirvetuximab Soravtansine (ELAHERE)	Disulfide-containing cleavable linker sulfo-SPDB	Glutathione cleavable	Maytansinoid DM4	ImmunoGen

* Following the request of U.S. FDA, belantamab mafodotin was withdrawn from the market based on the outcome of the DREAMM-3 phase III confirmatory trials.
